# Alpha-amylase 1A copy number variants and the association with memory performance and Alzheimer’s dementia

**DOI:** 10.1186/s13195-020-00726-y

**Published:** 2020-11-21

**Authors:** Elin Byman, Katarina Nägga, Anna-Märta Gustavsson, The Netherlands Brain Bank, Johanna Andersson-Assarsson, Oskar Hansson, Emily Sonestedt, Malin Wennström

**Affiliations:** 1 Clinical Memory Research Unit, Department of Clinical Sciences Malmö, Lund University, Inga Marie Nilssons gata 53, SE-214 28 Malmö, Sweden; 2 Department of Acute Internal Medicine and Geriatrics, Linköping University, Linköping, Sweden; 3 Memory Clinic, Skåne University Hospital, Malmö, Sweden; 4 Netherlands Institute for Neuroscience, Amsterdam, the Netherlands; 5 Department of Molecular and Clinical Medicine, The Sahlgrenska Academy at University of Gothenburg, Gothenburg, Sweden; 6 Nutritional Epidemiology, Department of Clinical Sciences Malmö, Lund University, Malmö, Sweden

**Keywords:** Alzheimer’s disease, Memory, Salivary alpha amylases, DNA copy number variation, Montreal cognitive assessment, Gender, Human brain

## Abstract

**Background:**

Previous studies have shown that copy number variation (CNV) in the alpha (α)-amylase gene (*AMY1A*) is associated with body mass index, insulin resistance, and blood glucose levels, factors also shown to increase the risk of Alzheimer’s dementia (AD). We have previously demonstrated the presence of α-amylase in healthy neuronal dendritic spines and a reduction of the same in AD patients. In the current study, we investigate the relationship between *AMY1A* copy number and AD, memory performance, and brain α-amylase activity.

**Methods and materials:**

The association between *AMY1A* copy number and development of AD was analyzed in 5422 individuals (mean age at baseline 57.5 ± 5.9, females 58.2%) from the Malmö diet and cancer study genotyped for *AMY1A* copy number, whereof 247 where diagnosed with AD during a mean follow-up of 20 years. Associations between *AMY1A* copy number and cognitive performance where analyzed in 791 individuals (mean age at baseline 54.7 ± 6.3, females 63%), who performed Montreal Cognitive Assessment (MoCA) test. Correlation analysis between α-amylase activity or α-amylase gene expression and *AMY1A* copy number in post-mortem hippocampal tissue from on demented controls (*n* = 8) and AD patients (*n* = 10) was also performed.

**Results:**

Individuals with very high ( ≥10) *AMY1A* copy number had a significantly lower hazard ratio of AD (HR = 0.62, 95% CI 0.41–0.94) and performed significantly better on MoCA delayed word recall test, compared to the reference group with *AMY1A* copy number 6. A trend to lower hazard ratio of AD was also found among individuals with low *AMY1A* copy number (1–5) (HR = 0.74, 95% CI 0.53–1.02). A tendency towards a positive correlation between brain α-amylase activity and *AMY1A* copy number was found, and females showed higher brain α-amylase activity compared to males.

**Conclusion:**

Our study suggests that the degree of α-amylase activity in the brain is affected by *AMY1A* copy number and gender, in addition to AD pathology. The study further suggests that very high *AMY1A* copy number is associated with a decreased hazard ratio of AD and we speculate that this effect is mediated via a beneficial impact of *AMY1A* copy number on episodic memory performance.

## Background

Salivary alpha (α)-amylase is an enzyme foremost found in the saliva, where it breaks down food polysaccharides such as starch. The degrading property, i.e., the activity of the enzyme, corresponds to the number of gene copies of the salivary α-amylase gene (*AMY1A*), which interestingly varies highly among individuals [1, 2]. The large variety has been evolutionary connected to the carbohydrate intake of different hunter and gather populations [1], which further suggests a role for *AMY1A* CNV in dietary and energy metabolism [3]. Indeed, several population-based studies have shown a relationship between *AMY1A* copy number and body mass index (BMI), insulin resistance, and blood glucose levels [4–11]. Falchi et al. were the first to report a link between *AMY1A* copy number and BMI by demonstrating an eightfold higher risk for obesity in individuals with more than 4 copies of *AMY1A* compared to individuals with more than 9 copies of *AMY1A* [7]. This study was followed up by studies performed in other research groups who found similar associations, e.g., that high *AMY1A* copy numbers lead to less obesity and lower BMI [5–7, 11–13] and an absence of obesity in Mexican children with very high *AMY1A* copy numbers (> 10) [5]. However, other studies report no association between *AMY1A* CNV and BMI [9, 14–16]. Individuals with high *AMY1A* copy numbers have further been shown to be less prone to develop insulin resistance and diabetes [4, 10] and have lower postprandial glycemic response [10], whereas other studies see no such associations [15, 17].

Although salivary α-amylase is foremost found in the saliva, it has been detected in several other organs including lung, heart, ovaries, and intestines [18]. The exact function within these organs is not well studied, but it is likely that it has a similar polysaccharide degrading role as in the saliva. We have previously, by the use of different methods (including immunohistological staining, RT-qPCR, protein assays, and activity assays), demonstrated the presence of α-amylase within the human hippocampus [19]. Interestingly, we further noted that immunostaining against salivary α-amylase revealed structures resembling neuronal synapses [19]. This finding is intriguing given that recent studies have demonstrated the presence of glycogen in neuronal synapses and moreover shown that degradation of this synaptic glycogen is crucial for hippocampal long-term potentiation, a fundamental mechanism in memory formation [20, 21]. It is thus tempting to speculate that the synaptic α-amylase has a role in the degradation of synaptic glycogen and thereby the formation of memories. Although this idea has to be proven in future studies, we found support for the hypothesis when we discovered a reduction (sometimes a complete loss) of hippocampal synaptic α-amylase immunoreactivity in patients with Alzheimer’s dementia (AD) [19]. Since AD is a neurodegenerative disorder leading to progressive cognitive decline with memory loss and disruption of daily life, we speculate that the loss of synaptic α-amylase can be involved in the memory decline seen in these patients. This idea further raises the question whether high *AMY1A* copy number and potentially thereby also higher brain α-amylase activity could protect against AD-specific changes. Of note, associations between AD and genetic variants of amylase genes have not been found in previous GWAS studies. However, although GWAS can be useful as a screening method to find genes implicated in disorders, it fails to detect alterations linked to CNV when the correlation between CNV and genetic variations of a gene is low, as in the case of *AMY1A* [14]. To investigate the potential impact of *AMY1A* CNV on AD onset, we conducted a study where we investigate the relationship between *AMY1A* CNV and clinically diagnosed AD, memory performance, and brain α-amylase activity.

## Material and methods

### Individuals included in the study

**Fig. 1:**
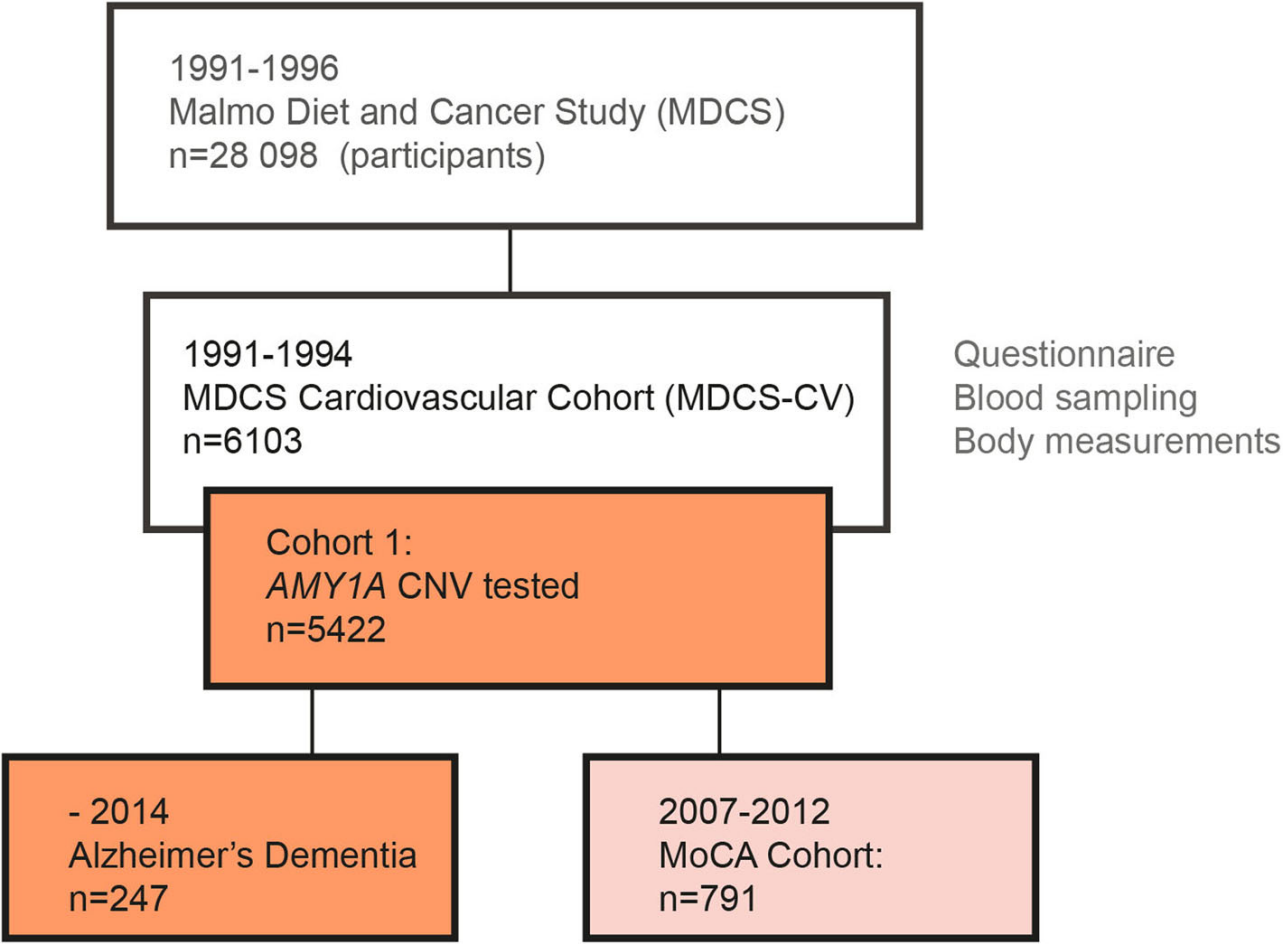
Flow diagram describing the study cohort 1. *AMY1A*, alpha-amylase 1 A gene; CNV, copy number variants; MoCA, Montreal Cognitive Assessment. MoCA testing was performed during reinvestigation of the MDCS cardiovascular cohort between 2007 and 2012

The study is performed on two cohorts. Cohort 1 includes individuals who participated in the Malmö Diet and Cancer Study (MDCS), which is a prospective population-based study where baseline examinations were performed between 1991 and 1996. The MSDC cohort consist of men and women born between 1923 and 1945 and 1923–1950, respectively whom been living in Malmö city. As many as 70,138 individuals were invited and finally 28,098 individuals participated in the study. The MDCS was approved by the ethical committee at Lund University (LU 51–90) and all participants provided written informed consent. At baseline, the participants filled out a questionnaire and had a clinical examination, blood samples were taken, and height, weight, and blood pressure were measured [22]. Half (50%) of the participants included in 1991 to 1994 were randomly selected for the cardiovascular sub cohort (*n* = 6103) [23]. Within the cardiovascular cohort, 5422 individuals (mean age at baseline 57.5 ± 5.9, females 58.2%) were genotyped for *AMY1A* copy number state. At a re-examination between 2007 and 2012, a sub fraction of 791 individuals (mean age at baseline 54.7 ± 6.3, females 63%) also completed the Montreal Cognitive Assessment (MoCA) (Table 3) [24]. Data on Alzheimer’s dementia diagnoses (*n* = 247, mean age at baseline 61.6 ± 4.4, females 67.2%) until December 31, 2014, were retrieved from the Swedish National Patient Register, and all diagnoses were reviewed and validated in medical records [25]. A flowchart of individuals enrolled in the study can be found in Fig. 1. Information about age, sex, *APOE* ε4 carrier status, BMI, education, fasting blood glucose, and diabetes were all retrieved from the MDSC and data collection procedures have previously been described [25, 26].

Cohort 2 consists of (*n* = 8) non-demented controls (NC) and (*n* = 10) AD patients (The Netherlands Brain Bank). Hippocampal gene expression and amylase activity in these individuals has previously been reported [19]. Written informed consent for the use of brain tissue and clinical data for research purposes was obtained from all patients or their next of kin in accordance with the International Declaration of Helsinki. Medical ethical evaluation committee of VU Medical Centre, Amsterdam, has approved the procedures of brain tissue collection, and the regional ethical review board in Lund has approved the study.

### Genotyping

Copy number state of *AMY1A* gene from individuals in the MDCS study was previously determined with droplet digital polymerase chain reaction (ddPCR) using a QX200 AutoDG Droplet Digital PCR system (Bio-Rad laboratories), described in Rukh et al. [9]. Quality control of the measurements was done by repeated runs for randomly selecting samples (~ 10%) as well as samples with high copy numbers. Determination of copy number state of *AMY1A* in individuals included in cohort 2 was performed on DNA extracted from the brain tissue. Briefly, the DNA was purified using QIAamp® DNA kit (Qiagen) according to the manufacturer’s instruction. The DNA concentration and purity were measured using Take 3 Micro-Volume plate and Eon spectrophotometer (Biotek, Winooski, VT, USA) and the DNA quality was evaluated with 1% agarose gel electrophoresis. The genotyping was performed by ddPCR at TATAA Biocenter (Gothenburg, Sweden) on a QX200 AutoDG Droplet Digital PCR system (Bio-Rad Laboratories) with *AP3B1* as the reference (2 copies). A negative control (no template) was included on each plate. Quality control and determination of copy number state was performed in Quantasoft version 1.7.4 (Bio-Rad Laboratories).

### α-Amylase gene expression and activity

The analysis of α-amylase gene expression in cohort 2 has been described previously [19]. In short, total RNA was purified from brain homogenates and thereafter converted into cDNA by reverse transcriptase. Reaction mixture (Thermo Fisher), probes (Thermo Fisher), and cDNA were mixed on a plate, and the real-time-qPCR analysis was performed using Viia 7 system (Applied Biosystems).

α-amylase activity measurement of hippocampal samples from cohort 2 was previously described in Byman et al. [19]. Briefly, brain samples were homogenized in amylase buffer (Abcam), and α-amylase activity was determined using Amylase assay kit colorimetric (Abcam) according to the manufacturer’s protocol. The absorbance was measured at 405 nm in a kinetic mode, and the increase/decrease of optical density at 405 nm during 30 min was then calculated (ΔOD405).

### Statistical analysis

**Fig. 2:**
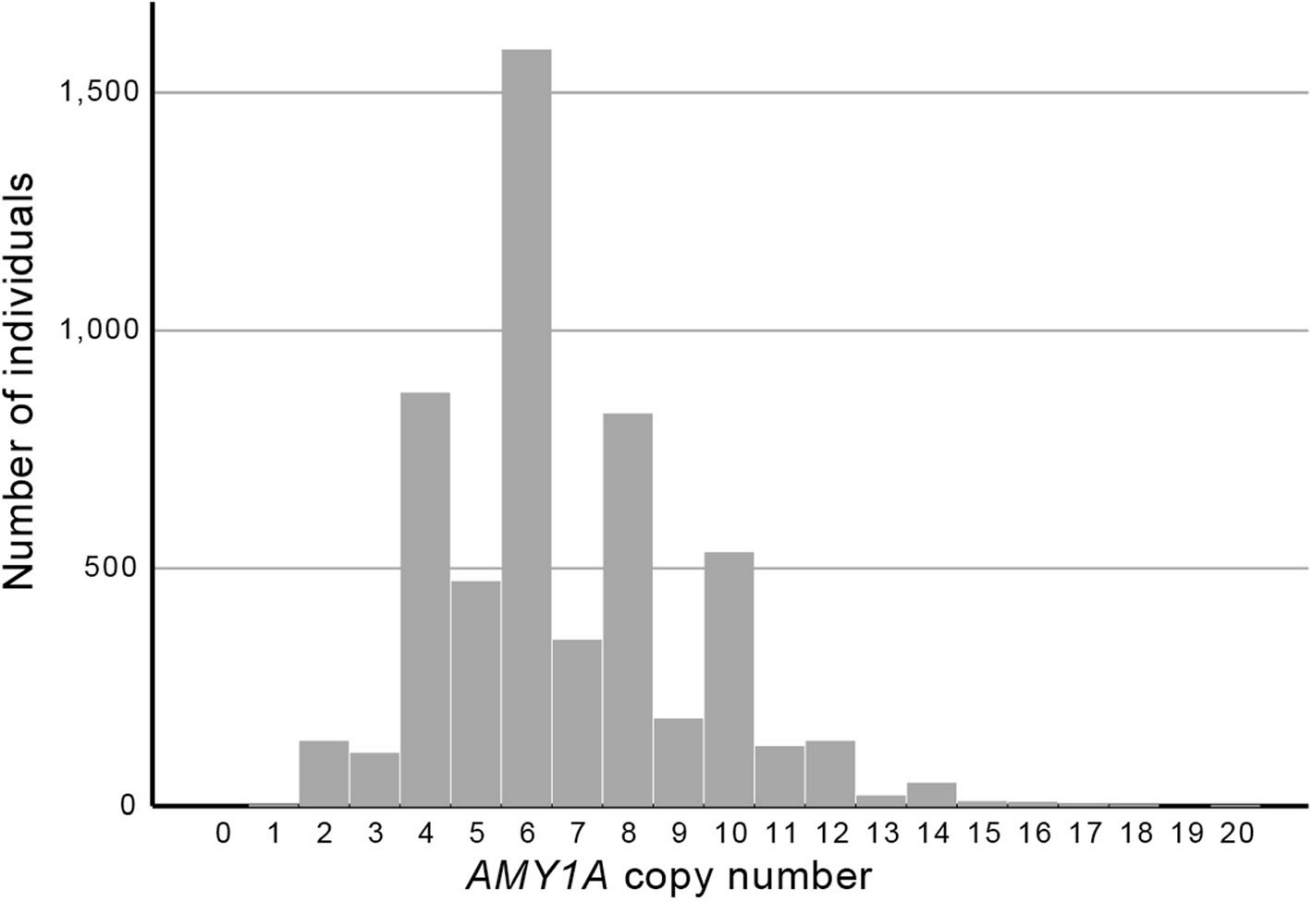
Histogram describing the distribution of *AMY1A* copy number between individuals in cohort 1

In cohort 1, *AMY1A* copy number (CN) were categorized into four groups; low (CN of 1–5), reference (CN of 6), high (CN of 7–9), and very high (CN of ≥ 10) in order to distinguish individuals with very high *AMY1A* copy numbers and form groups with roughly similar amount of participants. Individuals with an *AMY1A* copy number of 6 was set to be the reference group as this was the median number of copies and the most common *AMY1A* copy number in our cohort (see histogram in Fig. 2), which is in accordance with previously published *AMY1A* CNV studies [1, 5, 8, 13]. Statistical analysis was performed using SPSS software (version 25 for Mac, SPSS Inc., Chicago, IL, USA). *AMY1A* copy number in AD and individuals without AD was normally distributed (Kolmogorov-Smirnov test), and the difference between the two groups was analyzed using *t* test. Associations between *AMY1* copy number and development of AD were assessed with Cox regression models, both continuously per increase in copy number and categorically based on the four copy number groups. Death was treated as a competing risk event by censoring individuals at time of death (cause-specific hazard models). This approach was used since the research objective was etiological [27] and considering that 29% (1576/5422) of participants died during the 20-year follow-up. Individuals were also censored at the end of follow-up (December 31, 2014), if they were lost to follow-up (*n* = 49), or if they received another dementia diagnosis than AD (*n* = 166, e.g., vascular dementia, Lewy body dementia, or frontotemporal dementia). The hazard ratio (HR) of AD was thus estimated in individuals who were alive and not diagnosed with other dementia variants. Time was defined as years between baseline and event or censoring. The proportionality assumption was confirmed using Shoenfeld residuals (using R statistical software). We applied complete case analyses, thereby only including individuals with observed data on all entered variables in the models. Cox regression models were performed non-adjusted, adjusted for age, and fully adjusted (age, sex, education, BMI, *APOE* ε4, fasting blood glucose, and diabetes) and presented as hazard ratio (HR) with 95% confidences intervals (CI). Differences in MoCA test results between with low (1–5), reference (6), high (7–9), and very high (≥ 10) *AMY1A* copy number were analyzed non-adjusted using one-way ANOVA with Tukey post hoc test and fully adjusted using ANCOVA with covariates: age, sex, education, BMI, *APOE* ε4, fasting blood glucose, and diabetes. Normal distribution analysis of samples in cohort 2 was performed using Kolmogorov-Smirnov test. Since both copy number of *AMY1A* and α-amylase activity were not normally distributed, correlation analysis was performed using the Spearman correlation test and the difference in α-amylase activity between males and females was analyzed using the non-parametric Mann-Whitney test. Results are presented as median and range, and values of *p* < 0.05 were considered statistically significant; *p* values between 0.05 and 0.1 were considered to be a trend towards significance.

## Results

### Cohort 1—*AMY1A* copy numbers and hazard ratio for Alzheimer’s dementia

The descriptive statistics of the participants divided into four groups based on copy number are included in Table 1. No significant difference in *AMY1A* copy number was seen between individuals with and without AD using *t* test analysis (6.57 ± 2.16 vs 6.71 ± 2.48, *p* = 0.39). Cox proportional hazard analysis of AD during a 20-year follow-up showed significantly lower HR of AD in individuals with very high copy numbers of *AMY1A* (CN ≥ 10) compared to the reference group (CN = 6) (Table 2). When the analysis was adjusted for age and fully adjusted (age, sex, education, *APOE* ε4, BMI, and diabetes at baseline), the association remained significant (Table 2). Low copy numbers of *AMY1A* (CN = 1–5) showed lower HR of AD in both unadjusted and adjusted analyses, but the result was not significant. No association between *AMY1A* CNV and AD was found when copy number of *AMY1A* was modeled continuously (per 1 increase) (Table 2).

**Table 1:** Descriptive statistics of the study cohort stratified in four groups by numbers of α-amylase copies

	Alzheimer’s dementia (*n* = 247)			
	*AMY1* copy number			
**Baseline characteristics**	**1–5** **(*n*** **= 64, 26%)**	**6** **(*n*** **= 86, 35%)**	**7–9** **(*n*** **= 67, 27%)**	**≥ 10** **(*n*** **= 30, 12%)**
**Age, years**	61.6 (4.2)	61.6 (4.6)	61.3 (4.9)	62.4 (3.6)
**Women**	42 (66%)	64 (74%)	41 (61%)	19 (63%)
***APOE*** **ε4 carrier**	31 (48%)	59 (70%)	40 (61%)	18 (60%)
**Education:**				
**Primary/elementary school (≤ 8 years)**	31 (51%)	39 (46%)	32 (50%)	14 (50%)
**Body mass index**	26.4 (4.7)	25.3 (3.9)	26.3 (3.5)	26.8 (4.2)
**Fasting blood glucose, mmol/L**	5.2 (1.3)	5.2 (1.5)	5.3 (1.2)	5.0 (0.6)
**Diabetes at baseline**	3 (4%)	5 (6%)	10 (15%)	1 (3%)
**Diabetes, prevalent or incident**	11 (17%)	14 (17%)	20 (30%)	5 (17%)
	**Individuals without Alzheimer’s dementia (*n*** **= 5175)**			
	***AMY1*** **copy number**			
**Baseline characteristics**	**1–5** **(*n*** **= 1525, 30%)**	**6** (***n*** **= 1503, 29%)**	**7–9** **(*n*** **= 1289, 25%)**	**≥ 10** **(*n*** **= 858, 16%)**
**Age, years**	57.2 (5.9)	57.3 (5.9)	57.3 (5.9)	57.1 (6.0)
**Women**	868 (57%)	887 (59%)	727 (56%)	508 (59%)
***APOE*** **ε4 carrier**	428 (29%)	415 (28%)	359 (28%)	269 (32%)
**Education**				
**Primary/elementary school (≤ 8 years)**	670 (47%)	634 (45%)	577 (48%)	357 (44%)
**Body mass index**	25.6 (3.9)	25.9 (4.1)	25.7 (3.9)	25.8 (4.1)
**Fasting blood glucose, mmol/L**	5.2 (1.5)	5.2 (1.5)	5.1 (1.2)	5.2 (1.5)
**Diabetes at baseline**	78 (5%)	79 (5%)	55 (4%)	36 (4%)
**Diabetes, prevalent or incident**	340 (22%)	334 (23%)	254 (20%)	178 (21%)

Data is presented as mean (SD) or *n* (%). Missing data *APOE* (*n* = 90), education (*n* = 300), BMI (*n* = 7), and glucose (*n* = 544)

**Table 2:** Association between *AMY1* copy number and Alzheimer’s dementia during 20 years of follow-up

	Alzheimer’s dementia HR (95% CI)		
*AMY1* copy number	Unadjusted	Age-adjusted	Fully adjusted^1^
**Per 1 increase**	0.98 (0.93, 1.03)	0.98 (0.93, 1.03)	0.97 (0.92, 1.02)
**By four groups**			
**1–5**	0.74 (0.53, 1.02)	0.75 (0.54, 1.04)	0.75 (0.54, 1.05)
**6 (reference)**	1	1	1
**7–9**	0.93 (0.67, 1.27)	0.92 (0.67, 1.27)	0.86 (0.62, 1.20)
**≥ 10**	0.62 (0.41, 0.94)*	0.62 (0.41, 0.95)*	0.59 (0.38, 0.90)*
***n*** **events/total**	247/5422	247/5422	235/5028

Cox proportional hazards of Alzheimer’s dementia by number of copies of the *AMY1* gene

Data is presented as HR (95% CI) ^1^ adjusted for age, sex, education, *APOE* ε4, body mass index, and diabetes at baseline. *n* represents number of events (cases with AD) and total number of individuals included in the model

*p value < 0.05

### Cohort 1—association between *AMY1A* copy number and episodic memory performance

The descriptive statistics of the participants divided into four groups based on copy number are included in Table 3. One-way ANOVA with Tukey post hoc test analysis on MoCA total score did not show any significant difference between low (1–5), high (7–9), and very high copy number (≥ 10) and reference (6) copy number group respectively (Table 4), but when the analysis was fully adjusted, a trend towards a significant difference was seen between the groups (*p* = 0.09). One-way ANOVA on MoCA delayed word recall test showed on the edge to significant difference between the four copy number groups (*p* = 0.051). When the analysis was fully adjusted, the difference remained (*p* = 0.032). Further post hoc analysis showed a significantly higher (by 14%) test performance in individuals with very high *AMY1A* copy numbers compared to the reference group (*p* value = 0.045) (Table 4).

**Table 3:** Descriptive statistics of the study cohort stratified in quartiles by numbers of *AMY1A* copies

	MoCA test participants (*n* = 791)			
	*AMY1* copy number			
Baseline characteristics	1–5 (*n* = 245, 31%)	6 (*n* = 212, 27%)	7–9 (*n* = 205, 26%)	≥ 10 (*n* = 129, 16%)
**Age, years**	54.6 (5.4)	54.7 (5.3)	54.7 (5.2)	55.1 (5.1)
**Women**	152 (62%)	140 (66%)	131 (64%)	75 (58%)
***APOE*** **ε4 carrier**	72 (30%)	64 (31%)	58 (29%)	39 (31%)
**Education**				
**Primary/elementary school (≤ 8 years)**	93 (40%)	67 (33%)	77 (40%)	44 (35%)
**Body mass index**	25.1 (3.7)	25.3 (3.9)	25.4 (3.3)	25.0 (3.8)
**Fasting blood glucose, mmol/L**	4.9 (0.7)	5.0 (0.9)	4.9 (0.8)	5.0 (0.8)
**Diabetes at baseline**	6 (2%)	6 (3%)	6 (3%)	4 (3%)
**Diabetes, prevalent or incident**	47 (19%)	39 (18%)	30 (15%)	27 (21%)

Data is presented as mean (SD) or *n* (%). Missing data *APOE* (*n* = 17), education (*n* = 30), and glucose (*n* = 73)

**Table 4:** Associations between Montreal Cognitive Assessment test scores and AMY1A copy number variation

	MoCA test participants (*n* = 791) Mean ± SD	
*AMY1* copy number	Total MoCA score	Word recall score
**By four groups**		
**1–5**	25.5 (3.0)	3.0 (1.3)
**6 (reference)**^**1**^	25.1 (3.3)	2.8 (1.4)
**7–9**	25.5 (3.1)^a^	3.1 (1.4)
**≥ 10**	25.7 (3.1)	3.2 (1.2)*

Data is presented as mean (SD)

Data is analyzed using one-way ANOVA with Tukey post hoc test *p value < 0.05

^1^The most common AMY1A copy number variant is 6 and is therefore used as the reference group

^a^Missing data (*n* = 1)

### Cohort 2—brain α-amylase activity correlates with *AMY1A* copy number but is gender dependent

**Fig. 3:**
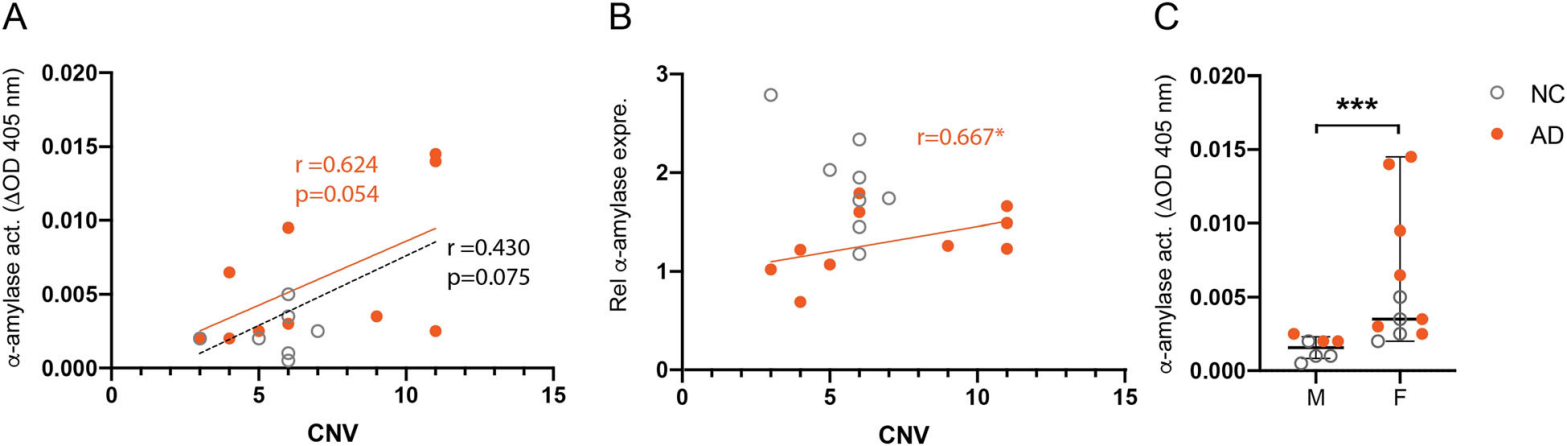
*AMY1A* copy number variation, alpha (α)-amylase activity and gene expression in post-mortem hippocampal tissue. Scatter plot in **a** shows a trend to correlation between *AMY1A* CNV and α-amylase activity (optical density (ΔOD) at 405 nm during 30 min) in the Alzheimer’s dementia (AD) group (orange) and a non-significant correlation in total cohort (dotted line) or non-demented control (NC) group (black). Scatter plot in **b** shows a significant correlation between *AMY1A* CNV and relative α-amylase expression normalized against values of housekeeping genes ribosomal protein L13A (*RPL13A*) and hydroxymethylbilane synthase (*HMBS*) in the AD group (orange), but no correlation was seen in the whole cohort or NC group (black). Column scatter plot in **c** showing α-amylase activity in AD patients (orange) and NC where median α-amylase activity in females is significantly higher compared to males. Data was analyzed using the Spearman (**a** and **b**) and Mann-Whitney *U* test (**c**) and presented as median and range (**c**). ***P < 0.001

To investigate whether *AMY1A* copy number corresponds to the overall α-amylase activity in the brain, we analyzed the association between *AMY1A* copy number and hippocampal α-amylase activity in a small number of individuals (*n* = 8 NC and *n* = 10 AD patients) in a second cohort. The demographics, cause of death, and neuropathological evaluation of the included individuals in cohort 2 are given in Table 5. The analysis showed a trend towards significantly positive correlation between the two variables (*r* = 0.430, *p* = 0.075). However, when the cohort where divided into NC and AD groups, we noted that the correlation was foremost seen in the AD group (*r* = 0.624, *p* = 0.054) (Fig. 3) and not in the NC group (*r* = 0.124, *p* = 0.769) (Fig. 3). Further analysis showed that *AMY1A* copy number did not correlate with α-amylase gene expression when the whole cohort was analyzed (*r* = 0.128, *p* = 0.613). Analysis on group basis revealed however a significant correlation between the two variables in the AD group (*r* = 0.667, *p* = 0.035) (Fig. 3b). Similar correlation was not detected in the NC group (*r* = − 0.546, *p* = 0.162) (Fig. 3b). Interestingly, we further noted higher hippocampal α-amylase activity in females compared to males (0.002 (0.0005 to 0.0025) vs 0.0035 (0.002 to 0.0145) Δ^30^OD^405^ *p* = 0.00044) (Fig. 3c), a finding detected also when the NC and AD groups were analyzed separately (0.003 (0.002 to 0.005) vs 0.001 (0.0005 to 0.002), *p* = 0.029, and 0.0065 (0.0025 to 0.145) vs 0.002 (0.002 to 0.0025), *p* = 0.017). However, neither copy number nor α-amylase gene expression differed between females compared to males (6.0 (3 to 11) vs 6.0 (3 to 11), *p* = 0.425, and 1.66 (0.69 to 2.79) vs 1.23 (1.02 to 2.03), *p* = 0.246, respectively). Additionally, we found no correlation between α-amylase activity and age, and the α-amylase activity did not differ in *APOE* ɛ4 carriers compared to *APOE* ɛ4 non-carriers. Finally, individuals with type 2 diabetes did not display altered α-amylase activity compared to non-type 2 diabetic individuals.

**Table 5:** Demographics of the included individuals in cohort 2

	Individuals in cohort 2 (*n* = 18)				
	Age ***Years***	Women	***APOE*** ε4 Carriers	Diabetes	CNV
NC (*n* = 8)	78.4 (13.5)	3 (50%)	3 (38%)	1 (13%)^1^	5.6 (1.2)
AD (*n* = 10)	84.5 (10.6)	7 (70%)	5 (50%)	8 (80%)	7.0 (3.2)

Data is presented as mean (SD) or *n* (%). ^1^Missing data *Diabetes n* = 1

*NC* non-demented controls, *AD* Alzheimer’s disease, *CNV* copy number variant

## Discussion

The aim of this study was to investigate whether *AMY1A* CNV was associated with clinically diagnosed AD, memory performance, and brain α-amylase activity. The result showed no difference in *AMY1A* copy number mean values between participants with AD and individuals without AD. However, individuals with very high *AMY1A* copy number had a significantly lower HR of AD compared to individuals in the reference group. A tendency towards a lower HR of AD was also observed in individuals with low *AMY1A* copy number compared to reference group. Moreover, the individuals with very high *AMY1A* copy number performed better on MoCA delay word recall test compared to individuals in the reference group, but no significant difference in total MoCA score was seen between the groups. Finally, α-amylase activity in human hippocampal tissue increased along with *AMY1A* copy number, but the association between the variables did not reach significance. Correlation between *AMY1A* CNV and α-amylase activity as well as α-amylase relative gene expression was foremost found in AD patients.

The observed lowered hazard of AD in combination with the better episodic memory performance in individuals with very high *AMY1A* CNV is intriguing, as it might suggest that high copy numbers of *AMY1A* could have a resilient impact on AD onset. Such resilience could be linked to the previously suggested association between *AMY1A* CNV and BMI, insulin resistance, and glucose homeostasis, factors also known to affect cognition [4, 7, 10]. However, since the result remained after full adjustment (where BMI, prevalence of type 2 diabetes, and fasting blood glucose levels were considered), it is likely that other mechanisms or factors are involved. Since we have previously discovered the presence of α-amylase in the brain, we find it tempting to speculate that production of α-amylase is one of these factors. How the very high *AMY1A* copy number variability (and potentially thereby very high production of brain α-amylase) can be implicated in the lowered AD risk and episodic memory remains to be investigated. But since α-amylase is known to efficiently degrade polysaccharides (such as glycogen) in the periphery, we find it likely that the enzyme has a similar role in the brain. The enzyme might thus be important for the degradation of glycogen in astrocytes and neurons [20, 28], which is known to be crucial for neurotransmitter production and memory formation [21, 29, 30]. The presence of α-amylase in activated astrocytes and neuronal synapses, found in our previous studies [19, 31], supports this idea.

Although a number of studies have demonstrated a clear correlation between *AMY1A* CNV and α-amylase expression as well as activity in saliva and plasma [1, 8], there are reports suggesting that other factors besides *AMY1A* CNV can influence the production as well [32]. Our correlations’ analysis of individuals included in cohort 2 showed only a tendency towards a significant correlation between *AMY1A* CNV and α-amylase activity, and we found significantly increased activity in female compared to males, despite the fact that the females in the cohort did not display higher copy numbers on average. These findings point towards a posttranslational gender-dependent regulation of the enzyme, an idea supported by a previous study demonstrating higher salivary α-amylase secretion in stressed female, but lower levels in stressed males [33]. Additionally, *AMY1A* copy number only correlates with α-amylase gene expression when the AD group was analyzed separately and not after analysis across the whole groups. This somewhat surprising result may be explained by the fact that AD patients displayed an overall lower α-amylase expression (regardless of *AMY1A* copy number), which in turn suggests AD pathology as an additional factor regulating α-amylase expression. Furthermore, the low copy number variation of *AMY1A* (a span from 3 to 7) within the NC group could influence the outcome of the analysis, which also might explain why the correlations between *AMY1A* copy number and α-amylase activity and expression is foremost pronounced in the AD group.

## Limitations

The are some limitations of this study, which needs to be addressed. First of all, the non-significant, but possibly lowered risk of AD in individuals with low *AMY1A* copy number indicates an almost U-shaped relationship between *AMY1A* copy number and risk of AD, which in turn could suggest that the finding is random. Since the cohort 1 moreover is rather small, it is important to verify the result in other larger cohorts, preferably with more AD patients. However, it should be stressed that the potential impact of *AMY1A* CNV on brain and peripheral energy metabolism is still largely unknown. We should therefore not rule out the possibility that low copy number of *AMY1A* under certain circumstances (such as environmental or dietary conditions) also can reduce the risk of AD. Studies demonstrating an impact of carbohydrate dietary on the association between *AMY1A* CNV and BMI [9] as well as increased glycemic response in individuals with high *AMY1A* copy number compared to low *AMY1A* copy number [15] highlight how complex the involvement of *AMY1A* CNV in energy metabolism is. The U-shaped relationship could also explain why we do not find significant differences between AD and individuals without AD. It should also be addressed that we have used register-based diagnoses, where structured dementia assessment was not performed on all participants. Since the diagnoses from clinical routine also may be less well characterized than diagnoses from a research protocol, we cannot rule out the possibility that some individuals with dementia were included as non-demented participants. Another limitation is the fact that we only have access to results from brief cognitive tests. These tests are designed to rapidly capture mild cognitive impairment in a clinical setting and do not distinguish different memory abilities like detailed neuropsychological tests do. Moreover, as the MoCA test is brief the scores can be fortuitous. The suggested association between high *AMY1A* CNV and episodic memory should therefore be re-examined in a cohort where an extended memory examination has been performed.

The CNV/activity study also has limitations, and the rather small cohort size (cohort 2) is one. Hence, the CNV/activity study needs to be verified in lager cohorts which include NC individuals with greater variation in *AMY1A* copy number. Moreover, the assay and RT-QPCR primers used in our study cannot distinguish between the different α-amylase isoforms. Hence, the *AMY1A* copy number in our analysis correlates with the combined activity and expression of all α-amylase isoforms present in the brain, which besides salivary α-amylase also includes pancreatic α-amylase (AMY2A) [31] (and potentially other unreported isoforms). Although studies have shown that individuals with high *AMY1A* copy number in general also have high *AMY2A* copy number [8], we cannot overlook the fact that the method limitation makes it harder to draw certain conclusions.

## Conclusion

To conclude, our studies suggests that individuals carrying a very high number of *AMY1A* gene copies (≥ 10) have a lower risk of AD and higher episodic memory capability. Since, brain α-amylase also appeared to increase (although significance was not reached) with increasing *AMY1A* copy number, we speculate that the beneficial impact seen in individuals with very high copy numbers can in part be due to a higher production or reserve of α-amylase in neuronal synapses of these individuals. The picture is however complex as the relationship between *AMY1A* CNV and lowered risk of AD tended to be U-shaped and we noted a gender-dependent and AD pathological impact on brain α-amylase, which stresses the need for further studies to dissect the role for α-amylase and their isoforms in development of AD.

## Data Availability

The data sets supporting the conclusions of this article can be made available upon request. MDCS data can be requested through an application to the MDCS steering committee.

## Abbreviations

AD: Alzheimer’s dementia
AMY1A: Salivary alpha-amylase
APOE ε4: Apolipoprotein ε4
BMI: Body mass index
CNV: Copy number variation
CN: Copy number
HR: Hazard ratio
MDCS: Malmö Diet and Cancer Study
MoCA: Montreal cognitive assessment
NC: Non-demented controls
